# Predicting resting energy expenditure among athletes: a systematic review

**DOI:** 10.5114/biolsport.2023.119986

**Published:** 2022-11-18

**Authors:** Diogo V. Martinho, Robert J. Naughton, Ana Faria, André Rebelo, Hugo Sarmento

**Affiliations:** 1 University of Coimbra, Research Unit for Sport and Physical Activity, Faculty of Sport Sciences and Physical Education, Coimbra, Portugal; 2 Polytechnic of Coimbra, Coimbra Health School, Dietetics and Nutrition, Coimbra, Portugal; 3 Laboratory for Applied Health Research (LabinSaúde), Coimbra, Portugal; 4 School of Human and Health Sciences, University of Huddersfield, Huddersfield, UK; 5 CIDEFES, Centro de Investigação em Desporto, Educação Física e Exercício e Saúde, Universidade Lusófona, Lisboa, Portugal; 6 COD, Center of Sports Optimization, Sporting Clube de Portugal, Lisbon, Portugal

**Keywords:** Calorimetry, Energy needs, Sports nutrition, Predictive equation, Basal rate

## Abstract

Resting energy expenditure (REE) is often estimated in athletes using equations developed from the general population however, the application in athletic-specific populations is questionable. The aim of this systematic review was to compare measured REE and estimations of REE obtained from non-sport participants and athletes. Inclusion criteria met PICO criteria: population – participants involved in organized sport; intervention – resting energy expenditure was obtained by calorimetry; comparator – equations to estimate REE; outcomes – comparisons between measured REE and predicted REE. The search was conducted in Web of Science all databases, PubMed and Scopus. Comparisons between measured REE and predicted REE as well the potential models to estimate REE developed among athletes were summarized. Allowing for variation among studies, equations developed within general populations were not comparable to REE measured by calorimetry in athletes. Equations across athletic samples were obtained but, few studies tested their validity across independent samples of sport participants. Nevertheless, equations developed within athlete populations seem to be widely unused in sports nutrition literature and practice. De Lorenzo and ten Haaf equations appear to present an acceptable agreement with measured REE. Finally, equations used among adults should not be generalised for youth sport participants.

## INTRODUCTION

The ability to estimate total energy expenditure (TEE) accurately is frequently desired by athletes and practitioners alike. Access to this information can help in the design of optimal fuelling strategies for training and competition, supporting training adaptation and performance [1]. There are three components of TEE: resting energy expenditure (REE), thermic effect of exercise and diet induced thermogenesis. Basal metabolic rate (BMR) and REE are often used as interchange terms but, represent different concepts. BMR is defined as the minimal amount of energy to maintain the vital functions such as respiration, heartbeat, normal body temperature while, REE represents the energy to maintain the body functions at rest. Briefly, the assessment of BMR requires more standardized conditions and it is more challenging to measure than REE [2]. REE among athletes needs particular attention given the substantial contribution of REE to determine TEE [3]. Additionally, REE has been used as a parameter to define energy deficiency in sport participants [4]. Considering the preceding, the measurement of REE needs to follow a standardized protocol. The REE is typically obtained during the morning from continuous measures of VO_2_ and VCO_2_ at rest and athletes are instructed to avoid exercise 12 hours before REE testing. The participant is positioned in the supine position for 30–45 minutes with a mask or mouthpiece attached and then, 5–10 additional minutes of VO_2_ and VCO_2_ measures are obtained to assess REE [2]. The mentioned protocol requires considerable equipment, time, exercise restriction and knowledge [2]. Therefore, indirect estimations of REE have been for non-sport participants [5] and athletes [3].

The Harris-Benedict [6] and Cunningham [7] equations emerged as potential predictive estimations of REE in athletes [1]. Additionally, metabolic active tissue, expressed by fat-free mass (FFM) or lean soft tissue (LST), accounted for 60–70% of REE [8] and by inference should be considered a key factor in estimation of REE. The Harris-Benedict equation [6] did not measure metabolic active tissues while in the Cunningham equation [7] lean body mass was estimated based on body mass and age. The Harris-Benedict equation was developed 124 years ago, in 239 healthy participants (136 males, 103 female) and incorporated age, stature and body mass as explanatory predictors [6]. In parallel, Cunningham reanalysed the data of 223 participants from Harris and Benedict [6] and excluded 16 trained athletes. In this equation, estimated lean body mass accounted for 70% of REE [7]. Interestingly, both equations are systematically used to estimate REE but, they are not specifically design for athletes. Consequently, the generalization and application of these equations among athletes are questionable.

The development and application of athletic-specific and sport-specific equations has not received much consideration within sports nutrition literature although it has been previously recognized that population specific estimations are needed [1]. Multiple equations to predict REE has been developed among athletes that participated in different sports [9–11]. Nevertheless, the validation of sport-specific equations to estimate REE in independent samples is lacking. Considering the contribution of REE to estimate TEE and the frequent use of equations validated in general population in sport participants, the aim of this systematic review is to compare estimated REE with measured REE in athletes. This review also summarized the models used in athlete populations.

## MATERIALS AND METHODS

The present systematic review followed the Cochrane guidelines [12] and it was conducted according to Preferred Reporting Items for Systematic Review (PRISMA) instructions [13].

### Eligibility criteria

The manuscripts included in the current systematic review followed PICO (population, intervention, comparator and outcome) criteria [12]: population comprised of participants involved in organized sport; intervention was defined as REE measured by calorimetry – requirements for REE assessment needed to be described; equations to predict REE were used as a comparator; outcomes described comparisons between REE measured and REE estimated or potential equations to predict REE; cross-sectional and cohort studies were included in this review. Published manuscripts or abstracts in English were considered for the present study. No filter was applied to year of publication. Manuscripts that did not presented descriptive statistics for REE were eligible to the review because provide qualitative information about the accuracy of equations. Authors of the papers included in the review were contacted where relevant data were not present within the manuscript.

### Information source and search strategy

Three electronic databases were consulted (i.e. Web of Science all databases, PubMed and Scopus) prior to 1^th^ January of 2022. The search strategy included the keywords: (“resting energy expenditure” OR “resting metabolic rate” OR “basal metabolic rate” OR “basal energy expenditure” OR REE OR “basal metabolism”) AND (“predictive equation*” OR “prediction equation*” OR equation* OR prediction*) AND (athlete* OR sport*). Potential search terms were identified taking into account previous words used in the titles, abstract and keywords. Two specialists (DVM/AF) developed the search strategy that was supervised by an experienced author in systematic reviews and meta-analysis (HS). Afterwards, a reference manager software (EndNoteTMX9, Clarivate Analytics, Philadelphia, PA, USA) was used to export the studies.

### Selection process

The initial screening by two independent authors (DVM and HS) according to the title and abstract. Then, full-text manuscripts were assessed to check if they met eligibility criteria. Discordances between authors were solved by consensus and if necessary a third independent reviewer (AF) was consulted.

### Data collection process

#### Data extrapolation

Two authors (DVM/HS) extracted the information from eligible studies. Data was organized and summarized on adapted template of Cochrane Consumers and Communication Review Group [14]. The list of parameters included in the previous spreadsheet were: (1) number of participants, (2) sport, (3) sex, (4) age, (5) competitive level, (6) measurement of REE, (7) equation studied, (8) potential independent variables, (9) statistical parameters about the model, (10) main findings, (11) limitations. Among adolescent Brazilian soccer players [15], means and standard deviations of WHO/FAO/ UNU, Harris-Benedict, Henry and Cunningham equations were calculated consulting the supplementary material from the original study.

#### Data Items

The main outcomes extracted were categorized in two different groups: (1) measured and predicted REE; (2) equation to estimate REE. Moreover, any equation to predict REE was contrasted with measured REE. The agreement of predicted REE was determined within 5% or 10% intervals of the measured REE. Since authors anticipated that few equations to predict REE were developed among athletes, predictive models of REE were extracted and summarized as an outcome domain.

#### Study risk of bias

According to a recent study [16], the Quality Assessment Tool for Observational Cohort and Cross-Sectional Studies developed jointly the National Heart, Lung and Blood Institute (NHLBI) and Research Triangle Institute International to examine individually the quality of studies [17] and was implemented in the current manuscript. The tool included fourteen questions and an overall approach (i.e. good, fair or poor). Items reflecting the following parameters: (1) research question; (2,3) study population; (4) groups recruited from the same population and uniform eligibly criteria; (5) sample size justification; (6) exposure assessed prior to outcome of measurement; (7) sufficient timeframe to see an effect; (8) different levels of the exposure effect; (9) exposures measurement; (10) repeated exposure assessment; (11) outcomes measurement; (12) blinding of outcomes assessors; (13) follow-up rate; (14) statistical analysis. Two independent observers completed the tool (DVM/HS) and possible disagreements were solved by a third reviewer (AF).

## RESULTS

### Study selection

**FIG. 1:**
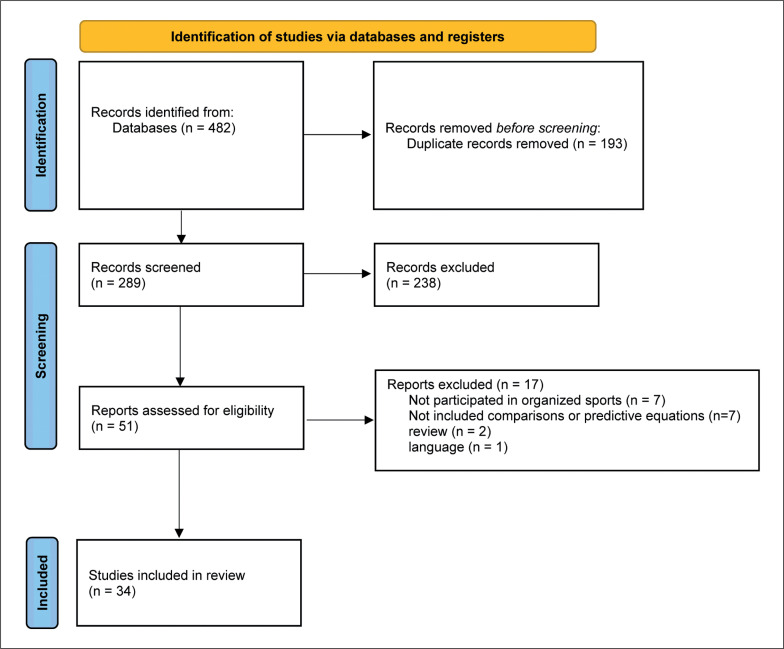
Identification of studies via databases and registers. *From:* Page MJ, McKenzie JE, Bossuyt PM, Boutron I, Hoffmann TC, Mulrow CD, et al. The PRISMA 2020 statement: an updated guideline for reporting systematic reviews. BMJ 2021;372:n71. doi: 10.1136/bmj.n71. For more information, visit: http://www.prisma-statement.org/

The databases searches identified 482 entries. Subsequently, duplicates were automatically and manually removed (n = 193). A total of 289 records were screened according to title and abstract, resulting in the exclusion of 238 records. The remaining 51 articles were read in full and 17 did not follow the eligible criteria: (1) the sample not clearly described as participants involved in organized sports (n = 7); (2) the manuscript did not show any comparison with equations or present a potential model for predicting REE (n = 7); (3) manuscripts were reviews (n = 2); (4) manuscript was not written in English (n = 1). Finally, 34 studies were selected to the current systematic review (Figure 1).

### Study characteristics

The characteristics of studies (sample, age, stature, body mass, FFM or LST) included in the present review are summarized separately by age group and sex in Supplementary Table 1. Twenty-one and seven studies included male adult [4, 9–11, 18–34] and youth sport participants [15, 35–40], respectively. Female adult athletes participated in 19 studies [4, 10, 18, 19, 21–25, 30–34, 41–45] while, five studies used samples of young athletes [35, 37, 38, 40, 46].

### Risk of bias in studies

Based on Quality Assessment Tool for Observational Cohort and Cross-Sectional Studies the risk of bias of studies was individually examined as shown in Supplementary Material 2. In general, studies did not estimate a priori sample size to examine differences between measured REE and estimated REE or to create an equation to extrapolate REE. In parallel, the inclusion criteria in each study were not described in some of the studies used in this systematic review. The overall quality rating of 28 studies was fair, 3 studies were classified as poor and 3 studies as good.

### Results of individual studies

The comparison of measured REE with predicted values was noted in twelves studies of adults [4, 18, 19, 20, 21, 22, 26, 27, 30, 41, 42, 43] and analysed in seven studies of young athletes [15, 35, 36, 37, 38, 39, 46] as shown in Table 1. Of interest, Harris-Benedict, Cunningham and WHO/FAO/UNU were the main equations examined. Contrasting findings across studies are notable. For example, among ultra-endurance [21] and high-levels athletes [22], Harris-Benedict tended to underestimate measured REE while among Royal ballet dancers [4] predicted REE was overestimated in comparison to measured REE. Regarding the Cunningham equation, it tended to overestimate measured REE in Olympic male and female athletes [18] and underestimate measured REE in ultra-endurance athletes [21]. Predicted REE by WHO/FAO/UNU equation was, on average, substantially less than measured REE among Indian male weightlifters [26] and it was considered the most appropriate estimation of REE amongst adult male soccer players from Malaysia [24]. Overall, the equations developed to predict REE in general population were not comparable to measured REE in athletic samples. The percentage of agreement reported in six studies [10, 18, 21, 23, 33, 35] was often less than 60% for Harris-Benedict [10, 18, 21, 22, 23, 33] and Cunningham [21, 22] equations in athletes. Although sport-specific equations to estimate REE had received less attention, an agreement > 60% [10, 23, 25] was obtained in three studies that used De Lorenzo et al. [9] equation (Table 2). Studies which presented equations to estimate REE are summarized in Table 3 [9–11, 20, 22, 23, 25–28, 31, 34, 36, 37, 40, 43–45]. Body weight and FFM emerged as the most determinant predictors of REE. Two studies used the sum of four [44] and seven [28] body compartments to calculate REE in 93 collegiate athletes and 10 sumo wrestlers, respectively. Recently, two equations included somatotype [22] and phase angle [11] as potential determinants of REE.

**TABLE 1:** Mean ± standard deviation of measured REE and predicted REE considering alternative equations presented separately for adult and young athletes.

Study	Sample	Sex					
		Male			Female		

		REE measured (kcal · day^−1^)	Equation	REE predicted (kcal · day^−1^)	REE measured (kcal · day^−1^)	Equation	REE predicted (kcal · day^−1^)
**ADULTS**							

Balci et al. [18]	Olympic athletes	1885 ± 323	Harris-Benedict	1864 ± 180	1361 ± 232	Harris-Benedict	1483 ± 143
			Mifflin-BMSA	1778 ± 138		Mifflin-BMSA	1425 ± 148
			Mifflin-FFM	1727 ± 149		Mifflin-FFM	1149 ± 111
			Schofield	1828 ± 186		Schofield	1466 ± 198
			Cunningham (1991)	1969 ± 167		Cunningham (1991)	1534 ± 124
			Owen	1644 ± 126		Owen	1325 ± 145
			Liu	1723 ± 180		Liu	1383 ± 184
			De Lorenzo	1911 ± 147		De Lorenzo	1597 ± 164
			Bernstein	1511 ± 154		Bernstein	1597 ± 128
			Nelson	1706 ± 201		Nelson	1339 ± 1635
			Johnstone	1838 ± 187		Johnstone	1229 ± 164
			Roza	1874 ± 85		Roza	1483 ± 69

Carlsohn et al. [19]	rowing and canoe racing	2675 ± 526	Harris-Benedict	2133 ± 188	1577 ± 253	Harris-Benedict	1737±200
			Cunningham (1980)	2260 ± 181		Cunningham (1980)	1734 ± 147

Cocate et al.[20]	cycling	2051 ± 169	Harris-Benedict	1699 ± 95			
			Schofield	1699 ± 85			
			FAO/WHO/UNU	1702 ± 85			
			Henry	1562 ± 76			

Devrim-Lanpir	endurance sports	2041 ± 301	Harris-Benedict	1701 ± 120	1788 ± 341	Harris-Benedict	1322 ± 82
et al. [21]			Mifflin	2038 ± 126		Mifflin	1602 ± 59
			Cunningham (1991)	1894 ± 141		Cunningham (1991)	1497 ± 61
			WHO/FAO/UNU – BMA	1726 ± 86		WHO/FAO/UNU – BMA	1321 ±37
			WHO/FAO/UNU – BM	1755 ± 84		WHO/FAO/UNU – BM	1388 ± 41
			Wang	1744 ± 157		Wang	1289 ± 68
			Sabounchi^1^	1743 ± 137		Sabounchi^1^	1363 ± 62
			Sabounchi^1^	1662 ±117		Sabounchi^1^	1383 ± 56
			Sabounchi^1^	1739 ± 89		Sabounchi^1^	1158 ± 30

Freire et al. [22]	high level athletes	2099 ± 400	Harris-Benedict	1896 ± 291	1577 ± 170	Harris-Benedict	1490±104
			ten Haaf – BM	2082 ± 258		ten Haaf – BM	1573 ± 155
			ten Haaf – FFM	2243 ± 326		ten Haaf – FFM	1695 ± 139
			WHO/FAO/UNU	1975 ± 302		WHO/FAO/UNU	1429 ± 132
			De Lorenzo	2046 ± 242		De Lorenzo	1683 ± 165
			Wong	1969 ± 262		Wong	1505 ± 127
			Jagim	2435 ± 392		Jagim	1645 ± 205
			Cunningham (1980)	2170 ± 309		Cunningham (1980)	1650 ± 132
			Cunningham (1991)	2039 ±309		Cunningham (1991)	1519 ± 132

Joseph et al. [26]	weightlifting	2217 ± 515	Katch-McArdle	1687 ± 198			
			Cunningham (1980)	1842 ± 202			
			WHO/FAO/UNU	1821 ± 226			
			ICMR	1727 ± 215			
			Harris-Benedict	1791 ± 221			
			Mifflin	1699 ± 172			
			Owen	1580 ± 148			
			Nelson	1294 ± 263			

Mackay et al. [41]	recreational and sub-elite athletes				1452 ± 267	Harris-Benedict	1438 ± 113
						Mifflin	1392 ± 140
						WHO/FAO/UNU	1460 ± 133

Mackenzie-Shalders et al. [27]	rugby	2389 ± 263	Cunningham	2287 ± 176			
			Harris-Benedict^2^	2242 ± 233			
			Harris-Benedict^2^	2213 ± 226			

Marques et al. [42]	karate				1689 ± 286	WHO/FAO/UNU	1401 ± 89
						Harris-Benedict	1449 ± 54
						Cunningham (1980)	1552 ± 122
						Henry	1326 ± 69

O'Neil et al. [43]	rugby				1651 ± 167	Cunningham (1980)	1665 ± 124
						Harris-Benedict	1545 ± 117
						ten Haaf – FFM	1690 ± 129
						ten Haaf – BM	1679 ± 166
						Jagim	1830 ± 219
						Watson – FFM	1520 ± 65
						Watson – BM	1623 ± 99

Sena et al. [30]	CrossFit	1885 ± 416	Harris-Benedict	1869 ± 188	1403 ± 258	Harris-Benedict	1397 ± 108
			WHO/FAO/UNU	1878 ± 154		WHO/FAO/UNU	1380 ± 105
			Henry	1708 ± 151		Henry	1307 ± 108
			Cunningham (1980)	2031 ± 165		Cunningham (1980)	1521 ± 126
			Cunningham (1991)	1873 ± 162		Cunningham (1991)	1373 ± 124
			Mifflin	1771 ± 147		Mifflin	1309 ± 164

Staal et al. [4]	ballet dancers	1692 ±103	Cunningham (1980)	1967 ± 104	1215 ±106	Cunningham (1980)	1504 ± 108
			Harris-Benedict	1896 ± 135		Harris-Benedict	1355 ± 127
			Koehler	1813 ± 73		Koehler	1378 ± 69

Tinsley et al. [31]	muscular physique	2337 ± 310^3^	Hayes	2166 ± 199	1566 ± 133^3^	Hayes	1438 ± 126
		2408 ± 350^3^	Cunningham (1980)	2245 ± 170	1633 ± 182^3^	Cunningham (1980)	1581 ± 107
			Cunningham (1991)	2083 ± 167		Cunningham (1991)	1432 ± 105
			Mifflin – FFM	1975 ± 152		Mifflin – FFM	1381 ± 96
			Mifflin – BM	1944 ± 144		Mifflin – BM	1396 ± 95
			Owen	2058 ± 172		Owen	1302 ± 96
			ten Haaf – FFM	2290 ± 176		ten Haaf – FFM	1604 ± 110
			ten Haaf – BM	2192 ± 168		ten Haaf – BM	1566 ± 112
			Harris-Benedict	2086 ± 176		Harris-Benedict	1454 ± 70
			WHO/FAO/UNU	2102 ± 160		WHO/FAO/UNU	1417 ± 77
			De Lorenzo	2032 ± 180		De Lorenzo	1677 ± 107

Watson et al. [45]	National Collegiate Athletic Association (NCAA) collegiate athletes				1466 ± 150	Harris-Benedict	1528 ± 98
						Schofield	1483 ± 132
						Mifflin	1472 ± 134
						Owen	1278 ± 64
						WHO/FAO/UNU	1496 ± 141
						Cunningham (1980)	1588 ± 129
						Taguchi	1366 ± 157

Wong et al. [24]	elite athletes	1715 ± 204	WHO/FAO/UNU	1690 ± 130	1384 ± 147	WHO/FAO/UNU	1311 ± 83
			Ismail	1461 ± 130		Ismail	1185 ± 72
			De Lorenzo	1734 ± 111		Cunningham (1980)	1451 ± 81
			Cunningham (1980)	1760 ± 163		Harris-Benedict	1387 ± 57
			Harris-Benedict	1684 ± 140			

**YOUTH**							

Cherian et al. [35]						IOM	1308 ± 63
	soccer	1343 ± 297	Cunningham (1980)	1375 ± 197	1135 ±117	Cunningham (1980)	1252 ± 83
			Henry	1428 ± 205		Henry	1262 ± 73
			Soares – BMA	1357 ± 124		Soares-FFM	1135 ± 80
			Soares – FFM	1252 ± 190		Patil-BM	1085 ± 74
			Patil – BM	1402 ± 137		Patil-BMSA	1100 ± 77
			Patil – BMSA	1184 ± 186		Wong	1317 ± 119
			De Lorenzo	1429 ± 223		ten Haaf:	1263 ± 8
			Wong ten	1334 ± 201			
			ten Haaf	1390 ± 204			

Hannon et al. [36]	soccer	1858 ± 215	Cunningham (1980)	1578 ± 281			
			De Lorenzo	1769 ± 263			
			Henry	1758 ± 272			
			Kim	1466 ± 191			
			Wong	1693 ± 193			

Loureiro et al. [38]	pentathlon	1559 ± 203	WHO/FAO/UNU	1679 ± 152	1357 ± 140	WHO/FAO/UNU	1376 ± 110
			Harris-Benedict	1610 ± 149		Harris-Benedict	1366 ± 89
			Henry	1667 ± 172		Henry	1279 ± 92
			Cunningham (1980)	1580 ± 171		Cunningham (1980)	1344±194

Łouszczki et al. [39]	soccer	1844 ± 328	Harris-Benedict	1513 ± 256			
			WHO/FAO/UNU	1567 ± 260			
			IMNA	1662 ± 303			
			Cunningham (1991)	1450 ± 264			
			Mifflin	1481 ± 224			
			Owen	1413 ± 147			
			Altman and Dittmer	1534 ± 283			
			Maffeis	1368 ± 150			
			Schofield	1589 ± 253			
			Molnar	1469 ± 239			
			De Lorenzo	1520 ± 298			

Kim et al. [37]	soccer	1648 ± 111	Harris-Benedict	1556 ± 58	1365 ± 186	Harris-Benedict	1418 ± 56
			WHO/FAO/UNU	1577 ± 65		WHO/FAO/UNU	1431 ± 63
			IMNA	1538 ± 70		IMNA	1367 ± 65
			Cunningham (1991)	1677 ± 95		Cunningham (1991)	1309 ± 58
			Mifflin	1543 ± 78		Mifflin	1342 ± 76
			Owen	1284 ± 38		Owen	1198 ± 37
			Altman and Dittmer	1867 ± 101		Altman and Dittmer	1640 ± 98
			Maffeis	1470 ± 60		Maffeis	1321 ± 58
			Schofield	1593 ± 63		Schofield	1431 ± 62
			De Lorenzo	1826 ± 99		De Lorenzo	1564 ± 97
			Park	1648 ± 51		Park	1590 ± 365

Oliveira et al. [15]	soccer	1717 ± 203	WHO/FAO/UNU	1854 ± 131			
			Harris-Benedict	1760 ± 126			
			Henry	1864 ± 148			
			Cunningham (1980)	1728 ± 129			

FAO/WHO/UNU (Food and Agriculture Organization/World Health/United Nations University); IMNA (Institute of Medicine of the National Academies); ICMR (Indian Council of Medical Research); IOM (Institute of Medicine); BMA (body mass, age); FFM (fat-free mass); BM (body mass); BMSA (body mass, stature, age).

1 Specific population-equation derived from meta-regression.

2 REE was estimated using different constants from Harris-Benedict equation.

3 REE was measured using two calorimetry devices.

**TABLE 2:** Percentage of agreement and disagreement between measured REE and predicted REE.

Study	n	equation	agreement	over-predicted	under-predicted
**ADULTS – MALE**					

Balci et al. [18]	25	Harris-Benedict	40%	36%	24%
		Mifflin-BMSA	40%	24%	36%
		Mifflin-FFM	60%	8%	32%
		Schofield	11%	28%	28%
		Cunningham	10%	52%	8%
		Owen	12%	4%	48%
		Liu	12%	12%	40%
		De Lorenzo	10%	40%	20%
		Bernstein	5%	0%	80%
		Nelson	15%	8%	32%
		Johnstone	13%	24%	24%
		Roza	6%	44%	32%

Devrim-Lanpir et al. [21]*	15	Harris-Benedict	20%	7%	73%
		Mifflin	47%	27%	27%
		Cunningham	47%	33%	20%
		WHO/FAO/UNU – BMA	20%	7%	73%
		WHO/FAO/UNU – BM	20%	7%	73%
		Wang	27%	7%	67%
		Sabounchi^1^	27%	7%	67%
		Sabounchi^1^	13%	7%	80%
		Sabounchi^1^	20%	7%	73%

Freire et al. [22]**	58	Harris-Benedict	36%		
		ten Haaf – BM	45%		
		ten Haaf – FFM	29%		
		FAO/WHO/UNU	33%		
		De Lorenzo	38%		
		Wong	29%		
		Jagim	7%		
		Cunningham (1980)	50%		
		Cunningham (1991)	50%		

Freire et al. [22]*	58	Harris-Benedict	67%		
		ten Haaf – BM	72%		
		ten Haaf – FFM	59%		
		FAO/WHO/UNU	64%		
		De Lorenzo	69%		
		Wong	64%		
		Jagim	24%		
		Cunningham (1980)	71%		
		Cunningham (1991)	78%		

Frings-Meuthen et al. [23]	79	Harris-Benedict	48%	0%	52%
		FAO/WHO/UNU	63%	0%	37%
		Muller	66%	6%	28%
		Muller-FFM	66%	1%	33%
		Cunningham	68%	25%	7%
		De Lorenzo	72%	10%	18%

ten Haaf and Weijs [10]*	53	Cunningham	84.9%		
		De Lorenzo	77.4%		

Van Grouwn etal. [33]*	16	Mifflin	56.3%		
		Harris-Benedict	43.8%		

**ADULTS – FEMALE**					

Balci et al. [18]	24	Harris-Benedict	50%	42%	8%
		Mifflin	71%	17%	13%
		Mifflin	58%	17%	25%
		Schofield	54%	38%	8%
		Cunningham	54%	38%	8%
		Owen	38%	58%	4%
		Liu	67%	21%	12%
		De Lorenzo	42%	58%	0%
		Bernstein	17%	8%	75%
		Nelson	33%	8%	58%
		Johnstone	54%	29%	17%
		Roza	38%	63%	0%

Devrim-Lanpir et al. [21]*	15	Harris-Benedict	13%	0%	87%
		Mifflin	53%	13%	33%
		Cunningham	20%	13%	67%
		WHO/FAO/UNU – BMA	13%	0%	87%
		WHO/FAO/UNU – BM	27%	0%	73%
		Wang	13%	0%	87%
		Sabounchi (2013)^1^	20%	0%	80%
		Sabounchi (2013)^1^	27%	0%	73%
		Sabounchi (2013)^1^	7%	0%	93%

Freire et al. [22]**	44	Harris-Benedict	30%		
		ten Haaf – BM	39%		
		ten Haaf – FFM	25%		
		FAO/WHO/UNU	25%		
		De Lorenzo	36%		
		Wong	36%		
		Jagim	39%		
		Cunningham (1980)	41%		
		Cunningham (1991)	43%		

Freire et al. [21]*	44	Harris-Benedict	59%		
		ten Haaf – BM	66%		
		ten Haaf – FFM	59%		
		FAO/WHO/UNU	57%		
		De Lorenzo	75%		
		Wong	68%		
		Jagim	59%		
		Cunningham (1980)	70%		
		Cunningham (1991)	73%		

Frings-Meuthen et al. [23]	34	Harris-Benedict	47%	3%	50%
		FAO/WHO/UNU	41%	6%	53%
[23]		Muller	47%	3%	50%
		Muller-FFM	46%	3%	52%
		Cunningham	64%	36%	0%
		De Lorenzo	62%	27%	12%

ten Haaf and Weijs [10]	37	Cunningham	78.4%		
		De Lorenzo	59.5%		

Van Grouwn et al. [33]*	17	Mifflin	82.2%		
		Harris-Benedict	52.3%		

**YOUTH**					

Cherian et al. [35]*	male	21	Cunningham	71.4%	
			Henry	57.1%	
			Soares – BMA	61.9%	
			Soares – FFM	42.9%	
			Patil – BMA	61.9%	
			Patil – BMSA	38.1%	
			De Lorenzo	61.9%	
			Wong	76.2%	
			ten Haaf	66.7%	

Cherian	female	19	Cunningham	42.1%	
			Henry	47.9%	
			Soares	94.7%	
			Patil – BMA	78.9%	
			Patil – BMSA	89.5%	
			Wong	21.1%	
			ten Haaf	42.1%	

* percentage of accurate REE predictions (within 10% of the measured REE);

** percentage of accurate REE predictions (within 5% of the measured REE).

1 Specific population-equation derived from meta-regression. BMA (body mass, age); FFM (fat-free mass); BMSA (body mass, stature, age); WHO/FAO/UNU (Food and Agriculture Organization/World Health). Sena et al. combined male and female CrossFit participants.

**TABLE 3:** Equations developed among athletes to estimate REE.

Study	sex	sample	Equation REE
Cocate et al. [20]	male	cycling	REE = -12888.2 + 485.846 × FFM – 3.7846 × FFM^2–^24.0092 × age

De Lorenzo et al. [9]	male	water polo, judo, karate	REE = -857 + 9.0 × body mass + (11.7 × stature

Freire et al. [22]	male and female	high level athletes	REE = 729.50 + 175.64 × sex – 7.23 × age + 15.87 × body mass + 1.08 × stature REE = -2688.12 + 521.08 × sex + 42.86 × age + 18.98 × body mass + 16.76 × stature + 85.47 × mesomorphy + 140.54 × endomorphy – 8.24 × body mass × sex + 1.53 × body mass × endomorphy – 0.65 × body mass × age

Frings-Meuthen et al. [23]	male and female	master athletic athletes	REE = -222.088 + 18.577 × FFM + 6.753 × FM + 23.910 × temperature + 78.479 × sex

Hannon et al. [36]	male	youth soccer	REE = 1315 + 11.1 × FFM REE = 1254 + 9.5 × body mass

Jagim et al. [25]	male and female	National Collegiate Athletic Association (NCAA) collegiate athletes	REE = 19.46 × body mass + 775.33 (males) REE = 21.10 × body mass + 288.6 (females)

Joseph et al. [26]	male	weightlifting	REE = -164.065 + 0.039 × LBM

Kim et al. [37]	male and female	youth soccer	REE = 502.7 + (8.6 × body mass) + (9.7 × VO_2max_) REE = 730.4 + 15 × FFM

Marra et al. [11]	male	elite athletes	REE = 17.2 × body mass – 5.95 × age + 748 REE = 16.3 × body mass + 95.4 × phase angle – 93

MacKenzie-Shalders et al. [27]	male	rugby	REE = 29.71 × LBM – 24.56 (beginning of pre-season) REE = 26.75 × LBM + 145.44 (prior to competition)

Midorikawa et al. [28]	male	sumo wrestlers	REE* = (13 × skeletal muscle mass) + (4.5 × adipose tissue mass) + (240 × brain mass) + (200 × liver mass) + (440 × kidney mass) + (440 × heart mass) + (12 × residual mass)

O’Neil et al. [43]	female	rugby	REE = 649.6 + 18.91 × FFM REE = 150.1–6.858 × age – 2.946 × stature + 11.21 × body mass

Reale et al. [40]	male and female	different sports	REE = body mass × 11.1 + stature × 8.4–339.7 (males) REE = FFM × 14.5 + FM + 8.6 + stature × 5.7–35.9 (males) REE = body mass × 11.1 + stature × 8.4–537.1 (females) REE = FFM × 14.5 + FM + 8.6 + stature × 5.7–203.9 (females)

Taguchi et al. [44]	female	collegiate athletes	REE = 17.8 × body mass + 243 REE = 26.9 × FFM + 36 REE* = (2.3 × body mass) + (4.5 × adipose tissue) + (13 × skeletal muscle) + (54 × residual mass)

ten Haaf and Weijs [10]	male and female	different sports	REE = 11.936 × body mass + 587.7 x stature – 8.129 × age + 191.027 × sex + 29.279 REE = 22.771 × FFM + 484.264

Tinsley et al. [31]	male and female	muscular physique	REE = 25.9 × FFM + 284 REE = 24.8 × body mass + 10

Watson et al. [45]	female	National Collegiate Athletic Association (NCAA) collegiate athletes	REE = 88.1 + 2.53 × stature + 8.42 × body mass + 19.46 × age REE = 120.81 + 4.88 × stature + 8.24 × FFM + 5.71 × age

Wong et al. [34]	male and female	elite athletes	REE = 669 + 13 × body mass + 192 × sex

REE (resting energy expenditure); FFM (fat-free mass); VO_2max_ (maximal oxygen uptake); LBM (lean body mass); FM (fat mass).

* These equations were developed by other authors.

## DISCUSSION

The aim of the present study was to review the agreement between measured REE and predicted REE using estimative equations. Additionally, the current systemic review summarized estimations of REE obtained using participants involved in organized sports. In general, across different samples of sport participants, measured REE was not comparable with REE predicted from equations developed in general population. Consistent results were noted among participants classified as overweight and obese [47] as well in healthy older adults aged ≥ 60 years [24]. Two equations, De Lorenzo et al. [9] and ten Haaf and Weijs [10], included athletes from different sports. Although few studies tested the precision of these equations, an agreement of 72% and 68% was noted with measured REE in male [23] and female [22] athletes, respectively. Therefore, the De Lorenzo and ten Haaf equations seems to be acceptable alternatives to estimate REE in athletes. Although Harris-Benedict and Cunningham equations were claimed to estimate REE among athletes, population-specific equations are needed [1].

Among 49 Turkish Olympic athletes differences between measured REE and predicted REE by Harris-Benedict equation were, on average, negligible however, only 40% and 50% of males and females, respectively, were within 10% of the measured REE [18]. Conversely studies using the Cunningham equation provided inconsistent results – underestimating REE in 83% of adolescent athletes aged 13–19 years [40] while, among 90 adult sport participants [10] an acceptable agreement between measured REE and predicted REE was reported in males (84.9%) and females (78.4%). Recently, the application of the Cunningham equation was recommended for use in female athletes but not be considered in males [24]. The equation explained 34% of variance in measured REE and an error 15% of with Cunningham model was noted among males [24]. In general, predicted REE by the Cunningham equation should not be generalized for athletic samples. This equation estimated lean body mass based on age and body mass [48]. Studies about REE estimation in athletes applied different methods to determine body composition. ten Haaf and Weijs [10] used air displacement plethysmography technique in 90 adult athletes while a recent study in Premier League soccer academy athletes used DXA methodology obtain FFM [36]. Considering the preceding, few studies that analysed Cunningham equation adopted the same methodology to estimate metabolic active tissues as original author [7]. The Cunningham equation was reviewed in 1991 but inconsistent results to predict REE were also noted [49].

The Mifflin equation [50] also emerged as a potential model to provide sex-specific estimates of REE in sport [51]. Fat mass and FFM were estimated from skinfold subcutaneous adipose tissue measurements [52, 53] and final sex-specific equations incorporated age, body mass and stature. The original sample included 247 females (ranging 20–76 years-old) and 251 males (age ranging 19–78 years-old). Of those, 112 females and 122 males were classified as obese [50]. Not surprisingly, predicted REE by the Mifflin equation tended to underestimate -114 kcal and -94 kcal measured REE among males and females CrossFit athletes, respectively [30]. In a sample of 9 power-lifters and 3 weightlifters Mifflin equation differed 11% of measured REE and it was supported that WHO/ ONU/UNU should be used to predict REE [29]. The sample of WHO/ ONU/UNU derived from the 7173 European and North American data points. Even though 3338 data points were obtained from active Italian participants with an elevated REE [54] this equation seems to be not applicable in athletes. Differences between measured REE and predicted REE using WHO/ONU/UNU equation ranged 466–287 kcal · day^−1^ in 30 ultra-endurance athletes aged 23–55 years [21]. Overall, the equations developed in general population should not be generalized for participants involved in organized sport. As a result, studies involving athletes proposed new predictive models to estimate REE.

Two potential equations to predict REE among athletes [9, 10] were compared with indirect calorimetry. Based on 126 male elite athletes from different sports minimal differences (21 kcal · day^−1^ and 60 kcal · day^−1^) were reported between the De Lorenzo et al. [9] and ten Haaf and Weijs [10] equations and measured REE [11]. The latter equation successfully predicted REE (within ± 10%) in 31 out of 36 female adult rugby players [43]. However, REE was underestimated by the De Lorenzo equation in young male soccer players [34, 36]. Three particular issues need highlighting: (1) De Lorenzo and ten Haaf models were validated in sport participants but received little consideration in sports nutrition literature and practice; (2) both equations combined adult athletes from different sports; (3) equations were developed in adults should not be generalized for youth sport participants.

Although the considerable number of studies extracted in the current review, a possible limitation is the inclusion of only English records. Additionally, grey literature was not also considered. Only one estimated a priori sample size necessary to create predictive models of REE. Future studies need to cross-validate the equations which used athletes from different modalities in larger sport-specific samples. Of note, predictions of REE only using female athletes are available in the literature and future research is required. The sex-specific equation proposed by ten Haaf and Weijs [10] is also adequate to predict REE in female athletes. The equation developed by De Lorenzo et al. [9] only included male participants from different sports hence, it is a valid alternative to estimate REE in male athletes. Specific equations were developed for youth involved in different sports [16] and soccer players [39, 44] thereby, should be adopted in studies of young athletes.

Findings of current review are crucial for nutritionists and/or staff providing nutrition support within sport in order to optimise total daily energy intake. The use of indirect equations in athletes, especially those that were developed in general population, tended to produce different values of measured REE which in turn has impact on TEE (obtained by multiplying REE and an appropriate physical activity factor). In summary, De Lorenzo et al. [9] and ten Haaf and Weijs [10] seem to be the most appropriate equations to predict REE among adult athletes and needed particular attention by sport nutritionists. Validation of predictive models to estimate REE required future research particularly in sport-specific samples and youth athletes.

## Supplementary Material

Predicting resting energy expenditure among athletes: a systematic review
