# Supplementary material for: Predicting resting energy expenditure among athletes: a systematic review
Source: Biol Sport. 2022 Nov 18;40(3):787–804. doi: 10.5114/biolsport.2023.119986 (PMC10286600; doi:10.5114/biolsport.2023.119986)
Supplement: Predicting resting energy expenditure among athletes: a systematic review [file JBS-40-119986-s1.pdf]

SUPPLEMENTARY TABLE 1. Summary of sample characteristics (sport, age, body size, body composition).

Study	Sport (n)	Age (years)	Stature (cm or m)	Body mass (kg)	Fat-free mass or lean soft tissue (kg or %)
		**MALE – ADULTS**			
Balci et al. [18]	Olympic athletes (n = 25)	19.1 ± 1.5	178.7 ± 6.1	75.4 ± 12.4	66.7 ± 7.6
Carlsohn et al. [19]	rowing and canoe racing (n = 8)	23.0 ± 5.0	193.0 ± 7.0	92.9 ± 10.0	81.0 ± 8.0 kg
Cocate et al. [20]	cycling (n = 15)	24.4 ± 3.7	174.7 ± 4.9	67.1 ± 5.5	62.0 ± 4.1 kg
De Lorenzo et al. [9]	water polo (n = 22), judo (n = 12), karate (n = 17)	22.3 ± 3.5	178.4 ± 7.1	78.0 ± 11.5	63.4 ± 6.6 kg
Devrim-Lanpir et al. [21]	triathlon (n = 10), ultra-marathon (n = 5)	38.4 ± 5.3	178.2 ± 7.4	73.0 ± 7.4	63.4 ± 6.4 kg
Freire et al. [22]	high level athletes (n = 58)	24.6 ± 3.8	182.6 ± 8.1	85.3 ± 20.1	77.2 ± 14.3 kg
Frings-Meuthen et al. [23]	master athletic athletes (n = 79)	57.1 ± 11.7	174.9 ± 7.2	74.2 ± 10.3	60.8 ± 8.4 kg
Jagim et al. [24]	football (n = 21), track and field (n = 4), baseball (n = 3)	20.3 ± 1.6	182.0 ± 6.1	94.5 ± 16.2	79.0 ± 7.7 kg
Jagim et al. [25]	National Collegiate Athletic Association (NCAA) collegiate athletes (n = 68)	20.1 ± 1.5	181.8 ± 5.9	93.7 ± 16.3	77.3 ± 8.1 kg
Joseph et al. [26]	weightlifting (n = 30)	21.5 ± 2.9	168.8 ± 6.3	76.0 ± 14.7	61.0 ± 9.2 kg
MacKenzie-Shalders et al. [27]	rugby (n = 18)	20.2 ± 1.7	184.0 ± 8.4	101.2 ± 14.5	81.3 ± 8.0 kg
Marra et al. [11]	elite athletes (n = 126)	26.9 ± 9.1	177.0 ± 7.0	71.3 ± 10.9	
Midorikawa et al. [28]	sumo wrestlers (n = 10)	19.4 ± 1.5	172.9 ± 8.4	109.1 ± 14.7	78.6 ± 0.7 kg
Moore et al. [29]	powerlifting (n = 9), weightlifters (n = 3)	22.4 ± 2.6	175.2 ± 7.8	92.0 ± 22.1	
Staal et al. [4]	ballet dancers (n = 20)	24.5 (21.0–28.5)	183.0 ± 4.4	72.8 ± 4.6	66.7 ± 4.7 kg
Sena et al. [30]	CrossFit (n = 52)	33.1 ± 5.7	1.75 ± 0.05	83.5 ± 12.3	69.6 ± 7.5 kg
ten Haaf and Weijs [10]	different sports (n = 53)	23.5 ± 5.0	1.72 ± 0.05	62.6 ± 6.6	78.4 ± 4.7%
Tinsley et al. [31]	muscular physique (n = 17)	26.0 ± 6.5	180.4 ± 7.2	94.0 ± 9.7	
Thompson and Manore [32]	endurance athletes (n = 24)	26.0 ± 4.0	177.2 ± 5.7	69.7 ± 7.6	63.4 ± 6.8 kg
Van Grouw et al. [33]	master athletes (n = 16)				
Wong et al. [34]	elite athletes (n = 92)	21.4 ± 3.0	170.6 ± 6.5	66.1 ± 8.5	57.1 ± 7.4 kg
		**MALE – YOUTH**			
Cherian et al. [35]	soccer (n = 21)	11.7 ± 2.1	160.0 ± 10.9	46.0 ± 11.1	39.8 ± 8.95 kg
Hannon et al. [36]	soccer (n = 99)	under-12: 12.3 ± 0.2	157.4 ± 4.1	45.5 ± 5.9	31.6 ± 4.2 kg
		under-13: 13.2 ± 0.2	162.7 ± 6.2	47.4 ± 5.6	34.6 ± 4.7 kg
		under-14: 14.3 ± 0.2	172.5 ± 8.0	56.9 ± 10.0	43.2 ± 8.9 kg
		under-15: 15.3 ± 0.3	175.9 ± 6.7	63.1 ± 7.1	49.3 ± 6.5 kg
		under-16: 16.4 ± 0.2	182.4 ± 5.8	72.9 ± 7.9	56.3 ± 5.3 kg
		under-18: 17.6 ± 0.7	182.7 ± 4.1	73.2 ± 8.1	57.9 ± 6.6 kg
		under-23: 19.9 ± 1.5	186.4 ± 6.0	80.3 ± 8.8	62.6 ± 5.9 kg
Kim et al. [37]	soccer (n = 30)	16.7 ± 1.0	176.9 ± 5.3	68.1 ± 5.3	60.5 ± 4.5 kg
Loureiro et al. [38]	pentathlon (n = 17)	15.0 ± 2.0	169.0 ± 7.0	58.5 ± 8.7	85.0 ± 3.4%
Łuszczki et al. [39]	soccer (n = 184)	13.2 ± 2.2	162.9 ± 14.9	52.4 ± 14.4	43.2 ± 12.0 kg
Reale et al. [40]	lacrosse (n = 6), basketball (n = 22), football (n = 14), baseball (n = 20), golf (n = 9), tennis (n = 11), track and field (n = 2), soccer (n = 13)	16.5 ± 1.5	179.0 ± 9.6	76.5 ± 16.6	60.7 ± 11.6 kg
Oliveira et al. [15]	soccer (n = 45)	15.69 ± 1.41	173.0 ± 7.5	67.6 ± 7.4	53.1 ± 6.0 kg
		**FEMALE – ADULTS**			
Balci et al. [18]	Olympic athletes (n = 24)	20.3 ± 2.1	163.3 ± 6.6	60.6 ± 12.7	47.0 ± 5.7 kg
Carlsohn et al. [19]	rowing and canoe racing (n = 9)	23.3 ± 3.0	175.0 ± 7.0	69.3 ± 11.0	56.1 ± 7.0 kg
Devrim-Lanpir et al. [21]	triathlon (n = 6), ultra-marathon (n = 9)	37.1 ± 7.9	162.7 ± 3.7	56.5 ± 4.1	45.3 ± 2.8 kg
Freire et al. [22]	high level athletes (n = 44)	25.7 ± 4.7	167.6 ± 8.0	64.3 ± 9.7	53.2 ± 6.1 kg
Frings-Meuthen et al. [23]	master athletic athletes (n = 34)	54.9 ± 11.6	165.0 ± 6.0	62.2 ± 10.0	48.2 ± 6.4 kg
Jagim et al. [24]	soccer (n = 15), swimming (n = 4), track and field (n = 3)	19.7 ± 1.4	166.5 ± 5.4	63.2 ± 7.3	49.2 ± 4.3 kg
Jagim et al. [25]	National Collegiate Athletic Association (NCAA) collegiate athletes (n = 48)	19.4 ± 1.3	166.5 ± 6.0	63.4 ± 12.7	48.7 ± 7.3 kg

SUPPLEMENTARY TABLE 1. Continue

Study	Sport (n)	Age (years)	Stature (cm or m)	Body mass (kg)	Fat-free mass or lean soft tissue (kg or %)
Mackay et al. [41]	recreational athletes (n = 12)	27.5 ± 12.3	169.3 ± 7.3	69.0 ± 9.4	
	sub-elite athletes (n = 13)	32.5 ± 7.4	167.9 ± 7.8	60.9 ± 6.7	
Marques et al. [42]	karate (n = 7)	21.7 ± 3.0	1.63 ± 0.04	62.1 ± 6.0	47.8 ± 5.6 kg
O'Neil et a. [43]	rugby (n = 36)	25.5 ± 4.8	167.5 ± 6.0	73.0 ± 10.4	53.0 ± 5.7 kg
Staal et al. [4]	ballet dancers (n = 20)	25.1 ± 4.8	170.4 ± 4.9	55.1 ± 5.4	45.6 ± 4.4 kg
Sena et al. [30]	crossfit (n = 90)	32.8 ± 6.8	1.63 ± 0.05	62.3 ± 9.4	46.4 ± 5.7 kg
Taguchi et al. [44]	collegiate athletes (n = 93)	20.3 ± 1.2	162.8 ± 6.4	57.0 ± 9.2	45.4 ± 6.2 kg
ten Haaf and Weijs [10]	different sports (n = 37)	23.1 ± 4.7	1.82 ± 0.09	75.7 ± 7.8	88.3 ± 4.9%
Tinsley et al. [31]	muscular physique (n = 10)				
Thompson and Manore [32]	endurance athletes (n = 13)	31.0 ± 5.0	162.5 ± 4.2	52.9 ± 5.6	45.1 ± 5.3 kg
Van Grouw et al. [33]	master athletes (n = 17)	25.8 ± 5.4	167.5 ± 5.7	63.8 ± 5.7	
Watson et al. [45]	National Collegiate Athletic Association (NCAA) collegiate athletes (n = 66)	19.7 ± 1.1	169.0 ± 9.0	67.3 ± 8.9	49.5 ± 5.8 kg
Wong et al. [34]	elite athletes (n = 33)	20.4 ± 2.1	160.7 ± 4.8	55.4 ± 5.7	43.2 ± 3.7 kg
**FEMALES – YOUTH**					
Branco et al [46]	gymnastics (n = 11)	16.6 ± 2.5	1.61 ± 0.06	53.3 ± 5.7	
Cherian et al. [35]	soccer (n = 19)	12.2 ± 1.8	153.6 ± 4.6	45.1 ± 6.6	34.2 ± 3.8 kg
Kim et al. [37]	soccer (n = 20)	16.4 ± 1.1	163.7 ± 5.2	56.1 ± 5.2	43.5 ± 2.7 kg
Loureiro et al [38]	pentathlon (n = 11)	14.0 ± 3.0	161.0 ± 6.0	52.6 ± 6.7	74.6 ± 9.2%
Reale et al. [40]	lacrosse (n = 3), basketball (n = 7), golf (n = 2), tennis (n = 4), track and field (n = 1), soccer (n = 11)	16.5 ± 1.2	166.8 ± 7.4	60.8 ± 10.2	43.6 ± 5.2 kg

SUPPLEMENTARY MATERIAL 2. Quality Assessment Tool for Observational Cohort and Cross-Sectional Studies to examine risk of bias for each manuscript.

Study	Balci et al. [18]	Branco et al. [46]	Carlsohn et al. [19]	Cherian et al. [35]	Cocate et al. [20]
1. Was the research question or objective in this paper clearly stated?	Yes	Yes	No	Yes	Yes
2. Was the study population clearly specified and defined?	Yes	Yes	Yes	Yes	Yes
3. Was the participation rate of eligible persons at least 50%?	NA	NA	NA	NA	No
4. Were all the subjects selected or recruited from the same or similar populations (including the same time period)? Were inclusion and exclusion criteria for being in the study prespecified and applied uniformly to all participants?	Yes	No	Yes	Yes	No
5. Was a sample size justification, power description, or variance and effect estimates provided?	Yes	No	No	No	No
6. For the analyses in this paper, were the exposure(s) of interest measured prior to the outcome(s) being measured?	NA	NA	NA	NA	NA
7. Was the timeframe sufficient so that one could reasonably expect to see an association between exposure and outcome if it existed?	NA	NA	NA	NA	NA
8. For exposures that can vary in amount or level, did the study examine different levels of the exposure as related to the outcome (e.g., categories of exposure, or exposure measured as continuous variable)?	NA	NA	NA	NA	NA
9. Were the exposure measures (independent variables) clearly defined, valid, reliable, and implemented consistently across all study participants?	Yes	Yes	Yes	Yes	No
10. Was the exposure(s) assessed more than once over time?	No	No	No	No	No
11. Were the outcome measures (dependent variables) clearly defined, valid, reliable, and implemented consistently across all study participants?	Yes	Yes	Yes	Yes	Yes
12. Were the outcome assessors blinded to the exposure status of participants?	NA	NA	NA	NA	NA
13. Was loss to follow-up after baseline 20% or less?	NA	NA	NA	NA	NA
14. Were key potential confounding variables measured and adjusted statistically for their impact on the relationship between exposure(s) and outcome(s)?	Yes	YES	Yes	Yes	Yes
Quality Rating (good, fair, poor)	Good	Fair	Fair	Fair	Fair

Study	De Lorenzo et al. [9]	Devrim-Lanpir et al. [21]	Freire et al. [22]	Frings-Meuthen et al. [23]	Hannon et al. [36]
1. Was the research question or objective in this paper clearly stated?	Yes	Yes	Yes	Yes	Yes
2. Was the study population clearly specified and defined?	Yes	Yes	Yes	Yes	Yes
3. Was the participation rate of eligible persons at least 50%?	NA	NA	NA	NA	NA
4. Were all the subjects selected or recruited from the same or similar populations (including the same time period)? Were inclusion and exclusion criteria for being in the study prespecified and applied uniformly to all participants?	No	Yes	Yes	Yes	No
5. Was a sample size justification, power description, or variance and effect estimates provided?	No	No	No	No	No
6. For the analyses in this paper, were the exposure(s) of interest measured prior to the outcome(s) being measured?	NA	NA	NA	NA	NA
7. Was the timeframe sufficient so that one could reasonably expect to see an association between exposure and outcome if it existed?	NA	NA	NA	NA	NA
8. For exposures that can vary in amount or level, did the study examine different levels of the exposure as related to the outcome (e.g., categories of exposure, or exposure measured as continuous variable)?	NA	NA	NA	NA	NA
9. Were the exposure measures (independent variables) clearly defined, valid, reliable, and implemented consistently across all study participants?	Yes	Yes	Yes	Yes	Yes
10. Was the exposure(s) assessed more than once over time?	No	No	No	No	No
11. Were the outcome measures (dependent variables) clearly defined, valid, reliable, and implemented consistently across all study participants?	Yes	Yes	Yes	Yes	Yes
12. Were the outcome assessors blinded to the exposure status of participants?	NA	NA	NA	NA	NA
13. Was loss to follow-up after baseline 20% or less?	NA	NA	NA	NA	NA
14. Were key potential confounding variables measured and adjusted statistically for their impact on the relationship between exposure(s) and outcome(s)?	Yes	Yes	Yes	Yes	No
Quality Rating (good, fair, poor)	Fair	Fair	Fair	Fair	Fair

Study	Jagim et al. [24]	Jagim et al. [25]	Joseph et al. [26]	Kim et al. [37]	Loureiro et al. [38]
1. Was the research question or objective in this paper clearly stated?	Yes	Yes	Yes	Yes	Yes
2. Was the study population clearly specified and defined?	Yes	Yes	Yes	Yes	Yes
3. Was the participation rate of eligible persons at least 50%?	NA	NA	NA	NA	NA
4. Were all the subjects selected or recruited from the same or similar populations (including the same time period)? Were inclusion and exclusion criteria for being in the study prespecified and applied uniformly to all participants?	Yes	No	No	Yes	Yes
5. Was a sample size justification, power description, or variance and effect estimates provided?	No	No	No	No	No
6. For the analyses in this paper, were the exposure(s) of interest measured prior to the outcome(s) being measured?	NA	NA	NA	NA	NA
7. Was the timeframe sufficient so that one could reasonably expect to see an association between exposure and outcome if it existed?	NA	NA	NA	NA	NA
8. For exposures that can vary in amount or level, did the study examine different levels of the exposure as related to the outcome (e.g., categories of exposure, or exposure measured as continuous variable)?	NA	NA	NA	NA	NA
9. Were the exposure measures (independent variables) clearly defined, valid, reliable, and implemented consistently across all study participants?	Yes	Yes	No	Yes	No
10. Was the exposure(s) assessed more than once over time?	No	No	No	No	No
11. Were the outcome measures (dependent variables) clearly defined, valid, reliable, and implemented consistently across all study participants?	NA	NA	NA	NA	Yes
12. Were the outcome assessors blinded to the exposure status of participants?	NA	NA	NA	NA	NA
13. Was loss to follow-up after baseline 20% or less?	NA	NA	NA	NA	NA
14. Were key potential confounding variables measured and adjusted statistically for their impact on the relationship between exposure(s) and outcome(s)?	Yes	Yes	Yes	Yes	Yes
Quality Rating (good, fair, poor)	Fair	Fair	Poor	Fair	Fair

Study	Łuszczki et al. [39]	Mackay et al. [41]	MacKenzie-Shalders et al. [27]	Marques [42]	Marra et al. [11]
1. Was the research question or objective in this paper clearly stated?	Yes	Yes	Yes	Yes	No
2. Was the study population clearly specified and defined?	Yes	Yes	Yes	Yes	No
3. Was the participation rate of eligible persons at least 50%?	NA	NA	NA	NA	NA
4. Were all the subjects selected or recruited from the same or similar populations (including the same time period)? Were inclusion and exclusion criteria for being in the study prespecified and applied uniformly to all participants?	Yes	Yes	No	No	No
5. Was a sample size justification, power description, or variance and effect estimates provided?	Yes	No	No	No	Yes
6. For the analyses in this paper, were the exposure(s) of interest measured prior to the outcome(s) being measured?	NA	NA	NA	NA	NA
7. Was the timeframe sufficient so that one could reasonably expect to see an association between exposure and outcome if it existed?	NA	NA	Yes	NA	NA
8. For exposures that can vary in amount or level, did the study examine different levels of the exposure as related to the outcome (e.g., categories of exposure, or exposure measured as continuous variable)?	NA	NA	Yes	NA	NA
9. Were the exposure measures (independent variables) clearly defined, valid, reliable, and implemented consistently across all study participants?	Yes	No	Yes	No	Yes
10. Was the exposure(s) assessed more than once over time?	No	No	Yes	No	No
11. Were the outcome measures (dependent variables) clearly defined, valid, reliable, and implemented consistently across all study participants?	Yes	Yes	Yes	Yes	Yes
12. Were the outcome assessors blinded to the exposure status of participants?	NA	NA	NA	NA	NA
13. Was loss to follow-up after baseline 20% or less?	NA	NA	No	NA	NA
14. Were key potential confounding variables measured and adjusted statistically for their impact on the relationship between exposure(s) and outcome(s)?	Yes	Yes	Yes	Yes	Yes
Quality Rating (good, fair, poor)	Good	Fair	Good	Fair	Fair

Study	Midorikawa et al. [28]	Moore et al. [29]	Oliveira et al. [15]	O'Neil et al. [43]	Reale et al. [40]
1. Was the research question or objective in this paper clearly stated?	Yes	Yes	Yes	Yes	Yes
2. Was the study population clearly specified and defined?	Yes	Yes	Yes	Yes	Yes
3. Was the participation rate of eligible persons at least 50%?	NA	NA	NA	NA	NA
4. Were all the subjects selected or recruited from the same or similar populations (including the same time period)? Were inclusion and exclusion criteria for being in the study prespecified and applied uniformly to all participants?	Yes	No	Yes	Yes	Yes
5. Was a sample size justification, power description, or variance and effect estimates provided?	No	No	No	No	No
6. For the analyses in this paper, were the exposure(s) of interest measured prior to the outcome(s) being measured?	NA	NA	NA	NA	NA
7. Was the timeframe sufficient so that one could reasonably expect to see an association between exposure and outcome if it existed?	NA	NA	NA	NA	NA
8. For exposures that can vary in amount or level, did the study examine different levels of the exposure as related to the outcome (e.g., categories of exposure, or exposure measured as continuous variable)?	NA	NA	NA	NA	NA
9. Were the exposure measures (independent variables) clearly defined, valid, reliable, and implemented consistently across all study participants?	Yes	No	Yes	No	No
10. Was the exposure(s) assessed more than once over time?	No	No	No	No	No
11. Were the outcome measures (dependent variables) clearly defined, valid, reliable, and implemented consistently across all study participants?	Yes	Yes	Yes	Yes	Yes
12. Were the outcome assessors blinded to the exposure status of participants?	NA	NA	NA	NA	NA
13. Was loss to follow-up after baseline 20% or less?	NA	NA	NA	NA	NA
14. Were key potential confounding variables measured and adjusted statistically for their impact on the relationship between exposure(s) and outcome(s)?	Yes	Yes	Yes	Yes	Yes
Quality Rating (good, fair, poor)	Fair	Poor	Fair	Fair	Fair

Study	Sena et al. [30]	Staal et al. [4]	Taguchi et al. [44]	ten Haaf et al. [10]	Thompson et al. [32]
1. Was the research question or objective in this paper clearly stated?	Yes	Yes	Yes	Yes	Yes
2. Was the study population clearly specified and defined?	Yes	Yes	Yes	Yes	Yes
3. Was the participation rate of eligible persons at least 50%?	NA	NA	NA	NA	NA
4. Were all the subjects selected or recruited from the same or similar populations (including the same time period)? Were inclusion and exclusion criteria for being in the study prespecified and applied uniformly to all participants?	Yes	No	No	No	Yes
5. Was a sample size justification, power description, or variance and effect estimates provided?	No	No	No	No	No
6. For the analyses in this paper, were the exposure(s) of interest measured prior to the outcome(s) being measured?	NA	NA	NA	NA	NA
7. Was the timeframe sufficient so that one could reasonably expect to see an association between exposure and outcome if it existed?	NA	NA	NA	NA	NA
8. For exposures that can vary in amount or level, did the study examine different levels of the exposure as related to the outcome (e.g., categories of exposure, or exposure measured as continuous variable)?	NA	NA	NA	NA	NA
9. Were the exposure measures (independent variables) clearly defined, valid, reliable, and implemented consistently across all study participants?	Yes	Yes	Yes	No	Yes
10. Was the exposure(s) assessed more than once over time?	No	No	No	No	No
11. Were the outcome measures (dependent variables) clearly defined, valid, reliable, and implemented consistently across all study participants?	Yes	Yes	Yes	Yes	Yes
12. Were the outcome assessors blinded to the exposure status of participants?	NA	NA	NA	NA	NA
13. Was loss to follow-up after baseline 20% or less?	NA	NA	NA	NA	NA
14. Were key potential confounding variables measured and adjusted statistically for their impact on the relationship between exposure(s) and outcome(s)?	Yes	Yes	Yes	Yes	Yes
Quality Rating (good, fair, poor)	Fair	Fair	Fair	Fair	Fair

Study	Tinsley et al. [31]	Van Grouw et al. [33]	Watson et al. [45]	Wong et al. [34]
1. Was the research question or objective in this paper clearly stated?	Yes	Yes	Yes	Yes
2. Was the study population clearly specified and defined?	Yes	No	Yes	Yes
3. Was the participation rate of eligible persons at least 50%?	NA	NA	NA	NA
o4. Were all the subjects selected or recruited from the same or similar populations (including the same time period)? Were inclusion and exclusion criteria for being in the study prespecified and applied uniformly to all participants?	Yes	No	No	Yes
5. Was a sample size justification, power description, or variance and effect estimates provided?	No	No	No	No
6. For the analyses in this paper, were the exposure(s) of interest measured prior to the outcome(s) being measured?	NA	NA	NA	NA
7. Was the timeframe sufficient so that one could reasonably expect to see an association between exposure and outcome if it existed?	NA	NA	NA	NA
8. For exposures that can vary in amount or level, did the study examine different levels of the exposure as related to the outcome (e.g., categories of exposure, or exposure measured as continuous variable)?	NA	NA	NA	NA
9. Were the exposure measures (independent variables) clearly defined, valid, reliable, and implemented consistently across all study participants?	Yes	No	Yes	Yes
10. Was the exposure(s) assessed more than once over time?	No	No	No	No
11. Were the outcome measures (dependent variables) clearly defined, valid, reliable, and implemented consistently across all study participants?	Yes	Yes	Yes	Yes
12. Were the outcome assessors blinded to the exposure status of participants?	NA	NA	NA	NA
13. Was loss to follow-up after baseline 20% or less?	NA	NA	NA	NA
14. Were key potential confounding variables measured and adjusted statistically for their impact on the relationship between exposure(s) and outcome(s)?	Yes	Yes	Yes	Yes
Quality Rating (good, fair, poor)	Fair	Poor	Fair	Fair
